# Estrogen Actions in Triple-Negative Breast Cancer

**DOI:** 10.3390/cells9112358

**Published:** 2020-10-26

**Authors:** Oliver Treeck, Susanne Schüler-Toprak, Olaf Ortmann

**Affiliations:** Department of Gynecology of Obstetrics, University Medical Center Regensburg, 93053 Regensburg, Germany; sschueler@caritasstjosef.de (S.S.-T.); oortmann@caritasstjosef.de (O.O.)

**Keywords:** triple-negative breast cancer, estrogen, estrogen receptor, G-protein coupled estrogen receptor 1, estrogen-related receptor

## Abstract

Triple-negative breast cancer (TNBC) lacks estrogen receptor (ER) α, but the expression of estrogen receptors ERβ and G protein-coupled estrogen receptor 1 (GPER-1) is able to trigger estrogen-responsivity in TNBC. Estrogen signaling in TNBC can also be activated and modulated by the constitutively active estrogen-related receptors (ERRs). In this review article, we discuss the role of ERβ and GPER-1 as mediators of E2 action in TNBC as well as the function of ERRs as activators and modulators of estrogen signaling in this cancer entity. For this purpose, original research articles on estrogen actions in TNBC were considered, which are listed in the PubMed database. Additionally, we performed meta-analyses of publicly accessible integrated gene expression and survival data to elucidate the association of ERβ, GPER-1, and ERR expression levels in TNBC with survival. Finally, options for endocrine therapy strategies for TNBC were discussed.

## 1. Triple-Negative Breast Cancer 

Breast cancer can be considered as a multifaceted disease including a heterogeneous group of tumors with great variety in clinical, morphological, and molecular aspects [1]. The established molecular classification of breast cancer is classically based on the expression of estrogen receptor α (ERα), progesterone receptors (PRs), and overexpression of the human epidermal growth factor receptor 2 (HER2); their presence has been assessed to predict prognosis as well as the potential response to targeted treatments [2,3].

**Figure 1:**
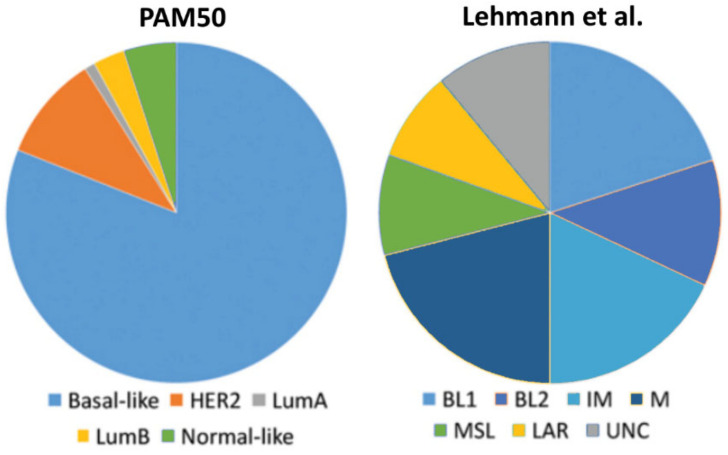
Comparison of the molecular subclassification systems applied to triple-negative breast cancers (TNBCs) by the PAM50 assay (Prosigna) and by Lehmann et al. [8]. The basal-like (BL) 1, BL2, immune-modulatory (IM) and mesenchymal (M) subclasses are preferentially of the PAM50 basal-like intrinsic subtype. UNC = unspecified.

About 15% of all breast cancer cases are triple-negative breast cancers (TNBCs), being responsible for more than 50% of breast cancer mortality [4,5]. This breast cancer subgroup lacks expression of ERα and PRs as well as HER2 amplification. TNBCs are more frequent in younger patients, and tumors are generally larger in size [6]. Moreover, TNBCs have a more aggressive clinical course than other forms of breast cancer, and are at the time of diagnosis usually of higher grade with frequent lymph node involvement [4,6,7]. Furthermore, as patients with TNBC do not benefit from targeted therapies directed against ERα such as tamoxifen or HER2 with trastuzumab, they have a poorer prognosis and a higher rate of distant recurrence than women with other breast cancer subtypes [6]. Less than one third of women with metastatic TNBC survive 5 years, and almost all die of their disease despite adjuvant chemotherapy [6]. However, TNBC is a highly diverse group of breast cancers. The gene expression cluster analysis identified various TNBC subtypes such as the basal-like (BL) 1, BL2, immune-modulatory (IM), luminal androgen receptor (LAR), mesenchymal (M), the mesenchymal/stem like (MSL) subtype, and the claudin low subtype [8]. Many of TNBCs are classified as basal-like either by immunohistochemistry or by correlation to the intrinsic molecular breast cancer subtypes [9,10,11]. Basal-like tumors express markers of the myoepithelium of the normal mammary gland, similar to the epidermal growth factor receptor (EGFR), p63, and the basal cytokeratins CK14, CK5/6 and CK17 [1,12]. TNBCs are likely to arise in BRCA1 mutation carriers and often have gene expression profiles similar to those of BRCA1-deficient tumors [7]. Comparison of the molecular subclassification systems applied to TNBCs reveals that the BL-1, BL2, immunomodulatory and mesenchymal TNBCs classified by Lehmann et al. [8] are preferentially of the basal-like intrinsic subtype of the PAM50 assay (Prosigna), that a large proportion of mesenchymal stem-like (MSL) TNBCs fit the intrinsic normal-like or claudin-low subtypes, and that the LAR subgroup corresponds in most part to the rare TNBCs classified by PAM50 as luminal or HER2-enriched (Figure 1).

## 2. Estrogen Signaling

Estrogens are well known to promote breast cancer growth primarily by activating ERα [13]. Classical, nuclear estrogen receptors such as ERα are ligand-activated transcription factors that mediate the effect of estrogens in the development and growth of both normal and malignant mammary tissues. Ligand-activated ERs are able to form dimers that, directly or through other proteins, bind specific estrogen response elements (EREs) in the target gene promoters and regulate their transcription [14,15]. ERα is a major driver of about 70% of breast cancers, and its role together with the ones of its target genes has been extensively studied. ERα and ERα-regulated genes represent the main targets in clinical approaches that aim to control hormonally responsive breast cancer [13]. Identification of the second nuclear estrogen receptor, ERβ, added a new level of complexity to estrogen signaling [16]. ERβ, coded by ESR2 gene, has functions and expression patterns distinct from ERα and is able to form homodimers or heterodimers with ERβ; its specific effect on gene regulation results from the modulation of ERα actions or by differential gene regulation in the absence of ERα. ERβ, like ERα, can modulate gene expression in a ligand-independent manner [17] or upon binding to its natural ligands (like 17β-estradiol, E2), but it also interacts with several synthetic agonists and antagonists [18]. Since most of ERβ target genes can also be regulated by ERα, when both receptors are co-expressed, the overall action of ERβ on the genome of hormone-responsive cells depends mainly on the relative concentration of both ERs in the cell [19]. In the normal mammary gland, ERβ is the most widely expressed ER, but its expression is known to decrease during malignant progression up to total absence in some invasive breast tumors [20]. Although the role of ERβ in breast cancer is still controversial mainly due to a multitude of IHC-based studies using unspecific antibodies, there is profound evidence both from in vitro and in vivo studies that this receptor has tumor-suppressive properties. In a recent study, knockout of the ESR2 gene in mice led to the formation of in situ ductal cancer in the prostate and mammary gland [21]. Various in vitro studies reported ERβ to suppress growth and invasion of breast cancer cell lines [22,23,24].

G-protein coupled estrogen receptor 1 (GPER-1) is a transmembrane receptor belonging to the family of G-protein-coupled receptors (GPCRs); it acts independently of estrogen receptors ERα and ERβ and is involved in rapid nongenomic effects of estrogen in normal and cancer tissue [25]. Estrogen binding to GPER-1 is able to activate the MAP kinase pathways by triggering release of heparin-binding EGF-like growth factor (HB-EGF), which in turn activates the epidermal growth factor receptor (EGFR) [26]. Estrogen binding to GPER-1 has also been reported to stimulate adenylyl cyclase and cAMP production, suggesting its coupling to Gαs, which in turn attenuates the EGFR-induced MAPK phosphorylation [27]. In addition, estrogen-mediated activation of c-fos transcription in breast cancer cells was also shown to occur in a GPER-dependent manner [28]. However, for some time there has been a lack of acceptance of GPER-1 being a true estrogen receptor, which was at least partially resolved by convincing reports of two independent research groups providing data demonstrating that E2 directly binds to GPER-1, which verified GPER-1 as a membrane-bound ER [29,30].

Estrogen-related receptors (ERR) are constitutively active orphan receptors that share a high degree of homology with the classical ERs. They do not bind estrogens but affect the estrogen response of breast cancer cells [31]. Generally, ERRs bind and regulate transcription via estrogen response elements (EREs) and extended ERE half-sites termed ERR response elements (ERREs) [32]. ERRs have been reported to affect ERα action [33,34]. ERRα has been shown to activate expression of the CYP19 aromatase, the enzyme responsible for the conversion of testosterone to estrogen [35,36]. Furthermore, ERRs can modulate a diverse array of genes involved in metabolism and physiology by its interaction with a wide variety of other nuclear receptors. ERRα, β, and γ are the proteins belonging to the family of ERRs known to exert distinct effects in breast cancer cells, which are topics of ongoing research.

## 3. Estrogen Actions in TNBC 

**Figure 2:**
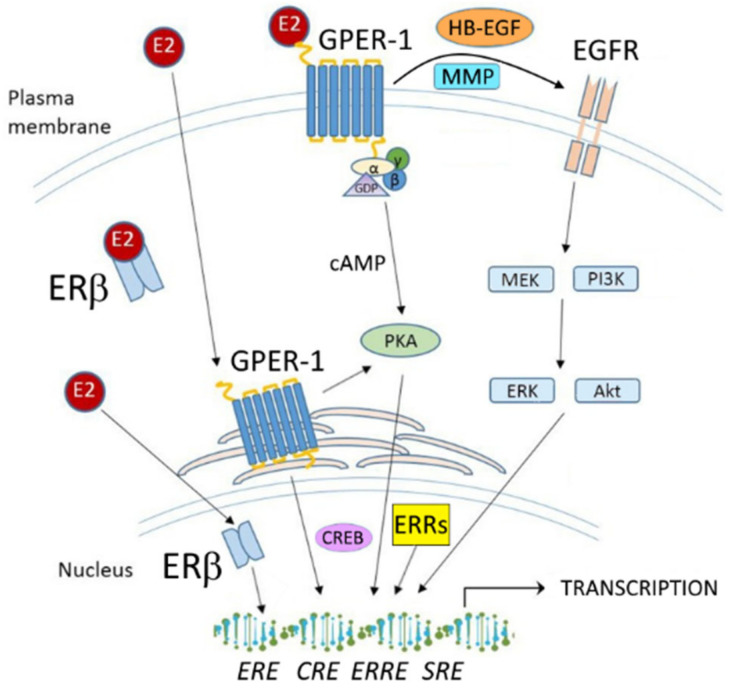
Schematic overview of estrogen signaling in TNBC cells. cAMP: cyclic adenosine monophosphate; PKA: protein kinase A; CREB: cAMP-response element binding protein; CRE: cAMP-response element; SRE: serum response element; MMP: matrix metalloproteinase; HB-EGF: heparin-binding EGF-like growth factor; EGFR: epidermal growth factor receptor; MEK: Mitogen-activated protein kinase kinase; ERK: extracellular signal-regulated kinase; PI3K: Phosphoinositide 3-kinase; AKT: Protein kinase B (PKB). Further abbreviations are addressed in the text.

Although TNBC cells do not express ERα, they are estrogen responsive via ERα-independent pathways. A recent study reported estrogens to promote the brain metastatic colonization of TNBC cells [37]. Using a TNBC experimental metastasis model, the study showed that ovariectomy decreased the frequency of brain metastases by 56% as compared to estrogen supplementation, and that the combination of ovariectomy and aromatase inhibitor letrozole further reduced the frequency of large lesions to 14.4% of the estrogen control. Furthermore, it was demonstrated [38] that increasing levels of circulating estrogens was sufficient to promote the formation and progression of ERα-negative cancers including TNBC, whereas the pharmacological inhibition of estrogen synthesis following pregnancy prevented ERα-negative tumor formation. Moreover, the effects of estrogen were shown to act via a systemic increase in host angiogenesis, in part through increasing mobilization and recruitment of bone marrow stromal derived cells into sites of angiogenesis and to a growing tumor mass. These observations suggest that estrogen may promote the growth of ERα-negative breast cancers like TNBC by also acting on cells distinct from the cancer cells to stimulate angiogenesis [38]. In contrast to ERα, the estrogen receptors β and GPER-1 as well as the estrogen-related receptors (ERRs) are frequently expressed in TNBC (Figure 2). In TNBC, E2-triggered effects are not only tumor-promoting but can also be antitumoral, for example by the activation of ERβ, which is reported to act as a tumor-suppressor. In the following part of this review, the role of these receptors as mediators or modulators of estrogen action in TNBC is discussed.

### 3.1. Estrogen Receptor β

The selective estrogen receptor downregulator (SERD) faslodex was recently reported to exert potent effects on ERβ-positive TNBC, notably suppressing TNBC cell growth in vitro and in vivo, and this effect was directly dependent on the level of intrinsically expressed ERβ, as depletion of this receptor inhibited faslodex action [39]. A multitude of studies suggest that ERβ is of major importance for estrogen action in TNBC [40,41,42,43,44,45,46,47,48]. ERβ is coded by the ESR2 gene in different splice variants, with the most studied forms being ERβ1 and ERβ2(cx), the two differing in the C-terminal region of the protein. Recently, the question of whether ERβ is expressed in normal breast or breast cancer at all, was raised in a comprehensive study reporting that only the rarely used monoclonal antibody PPZ0506 specifically targets ERβ in immunohistochemistry [49]. This study implied that numerous published studies with broadly accepted anti-ERβ antibodies have led to false positive results and thus have described ERβ expression incorrectly. In this study, using the mentioned antibody, no ERβ expression at all was detected in normal breast or breast cancer tissue but in testis and ovary. However, in a following study addressing this problem, two ERβ antibodies, PPZ0506 and PPG5/10, were demonstrated to specifically detect ERβ protein in Western blot and IHC analyses. Furthermore, using these antibodies, ERβ protein was shown to be highly expressed in normal human breast tissue as well as in 20%–30% of invasive breast cancers [50]. However, until this controversy is clarified in a reasonable manner, previous studies using invalidated antibodies at least have to be considered with caution.

The reported frequency of ERβ expression in TNBC ranges from 30% to 60%, with the latter rate being assessed in a study using the PPG5/10 antibody to examine 240 TNBC cases that were cytokeratin 5/6 and/or EGFR positive and thus were classified as basal-like [40,51]. Such frequency of ERβ expression suggests that this receptor is able to mediate estrogen actions in a considerable number of TNBC cases. Important insights into ERβ action in TNBC cells first came from in vitro studies employing TNBC cell lines like MDA-MB-231, MDA-MB-468, or Hs578T. It was reported that ERβ1 exogenous expression inhibits TNBC cell growth, thus arresting cell cycle at G1 phase, blocks cell colony formation and reduces tumor size in mice xenografts, effects that were enhanced by E2 treatment [41]. In this study, the question of ligand-independent ERβ effects was also addressed, showing that about 80% of the regulated genes were E2-dependent and only 20% ligand-independent. The growth-inhibitory effects of E2-bound ERβ in TNBC cells were reported to be due to inhibition of cyclin dependent kinases 1 and 7 and by regulation of genes involved in Wnt/β-catenin pathway (DKK1, WNT4, and CDH1) and in the G1/S cell cycle checkpoint control (CDKN1A), which are two signaling pathways well known for their role in cancer cell proliferation [41,42]. In a study from our lab, we could show that knockdown of ERβ significantly increased invasiveness of TNBC cells in vitro and upregulated expression of MMP13 and TNC genes, whereas activation of ERβ decreased TNBC cell invasiveness [43], with results being corroborated by a study using a different set of TNBC cell lines [44]. Action of ERβ in TNBC cells was recently demonstrated to depend on TP53 mutation status. In wild-type TP53-expressing cells, knockdown of ERβ increased apoptosis, whereas its overexpression resulted in increased proliferation. Opposite effects were observed in mutant TP53 TNBC cells, suggesting an important role of TP53 status in determining the function of ERβ [45]. In a recent study, ERβ was reported to inhibit proliferation of TNBC cell lines and to reduce expression of genes involved in angiogenesis, invasion, metastasis, and cholesterol biosynthesis, an oncosuppressive action mediated by association of ERβ with regulatory multiprotein chromatin remodeling complexes [46]. In a following study of this group, miR-181a-5p was found to be a mediator of ERβ-triggered inhibition of cholesterol biosynthesis in TNBC cells [47]. In a study on breast cancer stem cells (BSCs), ERβ was enriched in a notable number of basal-like and triple-negative BSCs but was significantly decreased after BSC differentiation, underscoring a tight connection between ERβ expression and the stem cell state. In triple-negative BCSs, treatment with ERβ antagonist PHTTP led to a decrease of mammosphere formation and tumor volume, suggesting that this receptor might be a potential target for a stem cell–specific therapy [52]. A recent study examined the role of ERβ in energy metabolism of TNBC cells and found that ERβ plays a critical role in maintaining mitochondrial function via transcriptional activation in mitochondria. Reduced mitochondrial ERβ expression resulted in the breakdown of mitochondrial activity in TNBC cells, thus increasing their proliferation via glycolysis resulting in tumor progression, whereas forced overexpression of mitochondrial ERβ reduced TNBC cell proliferation [53].

The results of most in vitro studies suggest tumor-suppressing effects of ERβ in TNBC cells, which are confirmed by studies on the prognostic significance of ERβ in TNBC. In a study including a cohort of 50 patients with TNBC, ERβ1 positivity was associated with a significantly higher disease-free survival (DFS) and overall survival (OS) rate at 5 and 10 years [48]. In a larger study including 571 patients with invasive TNBC, ERβ1 presence predicted significantly increased OS and DFS [40]. It was also demonstrated that ERβ1 can potentially interact with the PTEN/PI3K/pAKT pathway and that a ERβ1(+) / pAKT(−) status might predict the most favorable prognosis for TNBC. An opposite behavior was reported for the ERβ5 isoform, whose expression in TNBC was associated with a worse outcome in [54]. However, in a further study including 50 stage I-III TNBC cases, ERβ1 was found to be associated neither with OS nor with DFS [55].

**Figure 3:**
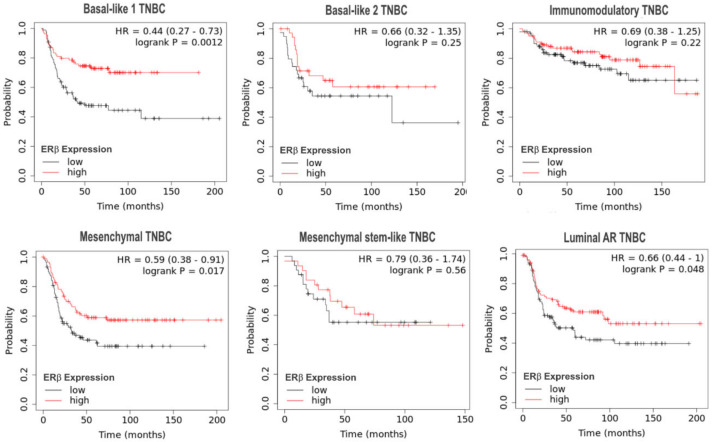
Kaplan–Meier analyses of the impact of ERβ mRNA expression on relapse-free survival (RFS) of patients with TNBC of the indicated 6 subtypes identified by Lehmann et al. [8] using the integrated microarray and clinical data KMplotter platform [56].

With regard to the 6 TNBC subgroups identified by Lehmann et al. [8], we performed a survival analysis of online integrated microarray and clinical data using the Kaplan–Meier plotter platform (https://kmplot.com) [56] to examine the impact of ERβ mRNA expression on relapse-free survival (RFS) of patients with the indicated subtypes of TNBC (Figure 3). In the TNBC subtypes basal-like 1, mesenchymal, and luminal androgen receptor (AR), higher levels of ERβ mRNA were associated with a significantly prolonged RFS. The other subtypes showed a weak trend towards prolonged RFS. When we analyzed breast cancer cases of the basal-like intrinsic subtype [12] using the same approach, we additionally observed a strong association of higher ERβ mRNA levels with prolonged RFS (HR = 0.64 (0.5–0.82), log rank *p* = 0.00051), with an upper quartile relapse-free survival of the low expression cohort of 17.76 months, compared to a survival of 30.6 months in the high expression cohort (data not shown). With regard to overall survival, no association with ERβ expression was observed in any of the examined subgroups, presumably due to the lower amount of subgroup survival data.

Confirming the importance of TP53 mutation status for ERβ action, a recent report demonstrated that mutant TP53-expressing TNBCs with high ERβ levels have a better survival than TNBCs without TP53 mutation. Furthermore, in TNBC cells with mutant TP53, tamoxifen increased the interaction between ERβ and mutant TP53, leading to the reactivation of TP73 and apoptosis. Considering that basal-like TNBC cases are enriched in TP53 mutations, these data suggest that the company of ESR2 with mutant TP53 not only can prognosticate TNBC patients, but more importantly help select a population for tamoxifen therapy [45].

BRCA1 mutations are prevalent in TNBC [57]. A large fraction of BRCA-associated TNBC express significant levels of ERβ [58]. Since the growth of BRCA1 mutant cells was found to be strongly inhibited by ERβ agonists, it was suggested that activation of ERβ by E2 or other agonists might be an interesting treatment option for TNBCs with BRCA1 mutations [59].

In summary, ERβ exerts the antitumoral actions in TNBC cells in vitro and the present reports and our bioinformatical survival analyses suggest that it also is associated with a longer OS and/or RFS in different types of TNBC. The action of ERβ in TNBC seems to be dependent on TP53 mutation status. In TNBC with high ERβ expression, presence of mutant TP53 does not only lead to beneficial survival, but it also seems to make these cells responsive to antiestrogen tamoxifen. However, further studies are necessary to confirm this link to tamoxifen efficacy as well as the use of faslodex or ERβ activation for TNBC therapy in the clinical situation.

### 3.2. G-Protein Coupled Estrogen Receptor-1

G-protein coupled estrogen receptor-1 (GPER-1) is expressed in the majority of TNBC cases [60,61]. First insights into the role of GPER-1 in TNBC came from in vitro studies. However, these studies were conflicting, as a part of them characterized this receptor as tumor-promoting, whereas others reported GPER-1 as a putative tumor-suppressor. This discrepancy might result from the GPER-1 agonist used, either the E2 or synthetic agonist G-1, which was shown to exert unspecific effects [62]. The first study suggesting GPER-1 to have tumor-promoting properties reported that knockdown of this receptor in GPER-1 expressing TNBC cells inhibited E2-induced proliferation, c-Fos expression, Src kinase activation, and EGFR transactivation, suggesting that GPER-1 is able to mediate growth-promoting E2 effects in TNBC cells [63]. In other studies of this group, treatment with estriol (E3) or EGFR inhibition by gefitinib was able to inhibit activation of TNBC cells triggered by GPER-1 mediated E2 action, and both experimental interventions were suggested to be potential therapy approaches for GPER-1 expressing TNBC [64,65]. In line with this, another group reported the estrogen-mediated nongenomic ERK signaling activated by GPER-1 to be involved in cell viability and motility of TNBC cells. Treatment with 17β-estradiol (E2) or tamoxifen (TAM) led to rapid activation of p-ERK1/2. Moreover, estrogen/GPER/ERK signaling was involved in increasing cell growth, survival, and migration/invasion by upregulating the expression of cyclin A, cyclin D1, and c-Fos [66]. Recently, NHERF1 was identified as a novel GPER-1 interacting protein that was reported to inhibit GPER-1 mediated TNBC cell proliferation and phosphorylation of ERK1/2 and Akt, and the loss of NHERF1 was suggested to play a pivotal role in the early stage of TNBC carcinogenesis [67]. As an additional interactor with GPER-1, focal adhesion kinase (FAK) was identified, demonstrating that estrogenic GPER-1 action leads to FAK phosphorylation in TNBC cells, and that in turn FAK inhibition prevents the migration of TNBC cells upon GPER activation [68].

With regard to studies suggesting a tumor-suppressive role of GPER-1 in TNBC, one report claimed GPER-1 agonist G-1 to inhibit TNBC cell growth via induction of cell cycle arrest in the G2/M phase, enhanced phosphorylation of histone H3, and caspase-3-mediated apoptosis [69]. In another study using G-1 as GPER-1 agonist, GPER-1 activation was found to inhibit EMT and metastasis of TNBC cells via NF-κB signaling [70]. A further report on G-1 triggering GPER-1 activation observed that after the trigger, there is a significant inhibition of interleukin 6 (IL-6) and vascular endothelial growth factor A (VEGF-A), resulting in the suppression of migration and angiogenesis of TNBC [71]. A recent in vitro study reported estrogens to inhibit VEGF expression and angiogenesis in TNBC by activating GPER-1. Moreover, E2 binding to GPER-1 inhibited in vivo tumor growth and angiogenesis and reduced the expression levels of VEGF, NF-κB/p65, STAT3, and the endothelial marker CD34 in TNBC cell xenograft tumors [72]. Finally, a very recent report demonstrated the activation of GPER-1 by E2 or G-1 to inhibit TNBC cell viability, proliferation, migration, invasion, angiogenesis, and EMT process via the CD151/miR-199a-3p bio-axis [73].

In line with the conflicting in vitro studies, the clinical relevance of GPER-1 in TNBC also remains controversial. In a study including 249 TNBC cases, high GPER-1 expression in TNBC was found to be associated with significantly shorter OS and PFS of premenopausal, but not postmenopausal patients [60]. In line with this, a small study including 48 patients reported recurrence at a mean follow-up of 36 months of 22.2% in the GPER-1-positive group and 9.5% in the GPER-1-negative group [61]. A conflicting study on 135 TNBC patients reported a significant association of high GPER-1 expression with longer OS and a negative association with high-grade tumors and lymph node metastasis [70].

When we performed a survival analysis with regard to the 6 TNBC subgroups identified by Lehmann et al. [8], using online available microarray and clinical data using the Kaplan–Meier plotter platform (https://kmplot.com) [56] to examine the impact of GPER-1 mRNA expression on relapse-free survival (RFS) or overall survival (OS) of patients with these subtypes of TNBC, we did not find any association with patients’ survival (data not shown). Analyzing GPER-1 mRNA levels in the intrinsic subtype of basal-like cancers [1], no association with survival was observed either.

In conclusion, further studies on the protein level are needed to unravel the present discrepancy on the role of GPER-1 in TNBC.

### 3.3. Estrogen-Related Receptors (ERRs)

ERRs are members of the nuclear hormone receptor super family of transcription factors and are classified as orphan receptors not being able to bind estrogens. However, recent studies demonstrated that they are not only able to regulate estrogen signaling by constitutive activity, but also to bind compounds with estrogen-like structures [74]. ERRs can modulate the expression of the ER-regulated transcriptome due to the high degree of structural similarity in the DNA binding domain [75].

ERRα expression shows a strong inverse relationship with ERα functionality in breast cancer [76]. In the absence of ERα, like in TNBC, ERRα becomes a major regulator of genes containing estrogen response elements (EREs), acting constitutively because it functions independently of estrogen [77]. ERRα was described as a metabolic regulator of energy homeostasis, and it was found to be associated with an increased risk of recurrence and adverse clinical outcomes of breast cancer patients in an ER-status independent manner [78]. The ChIP-chip analyses of breast cancer cells revealed that the majority of the genes regulated by ERRα are distinct from those controlled by ERs [79]. Recent studies suggest that ERRα functions as a transcriptional metabolic regulator and that it also promotes cancer development [80]. In breast cancer, increased ERRα levels were reported to associate with a ERα-negative and PR-negative tumor status [76]. With regard to TNBC, ERRα expression was reported to indicate worse prognosis and correlated with poor outcome predictors in TNBC and ERα-negative patients but not ERα-positive ones. However, in tamoxifen-treated TNBC patients, an improved outcome was observed with high ERRα expression [81]. In vitro studies support the oncogenic role of ERRα in TNBC. A recent study revealed that inhibition of ERRα can suppress the metastasis of TNBC cells via directly targeting fibronectin [82]. Limited studies are available examining the role of ERRβ in TNBC. ERRβ levels in breast cancer was shown to be relatively low in comparison to other ERRs [76]. Even more, ERRβ expression has been reported to be significantly lower in TNBC than in other breast cancer subtypes [83], suggesting that this orphan receptor might not play a major role in TNBC. However, the ERRβ gene ESRRB codes for at least three splice variants that were shown to have different transcription factor activity in basal-like versus other TNBC subtypes. The variants ERRβ2 and ERRβsf are reported to be broadly expressed in breast tumors at the protein level [83].

ERRγ, unlike ERRα, showed potential as a biomarker of favorable clinical course and hormonal sensitivity in invasive breast cancer. ERRγ was shown to be overexpressed in 75% of all breast cancer cases, resulting in the median ERRγ level being elevated in breast tumors compared with normal mammary epithelial cells. ERRγ overexpression is associated with a hormonally responsive positive ERα- and PR- status [76]. In TNBC, no significant ERRγ overexpression was observed. With regard to TNBC, an in vitro study demonstrated that one of the most ubiquitous endocrine disruptors, bisphenol A (BPA), increased the migration and invasion of TNBC cells via ERRγ by upregulation of matrix metalloproteinases (MMPs) 2 and 9 and the activation of ERK1/2 and Akt in TNBC cells [74]. BPA treatment could significantly increase the mRNA and protein expression of ERRγ, but not ERRα or ERRβ, in TNBC cells.

**Figure 4:**
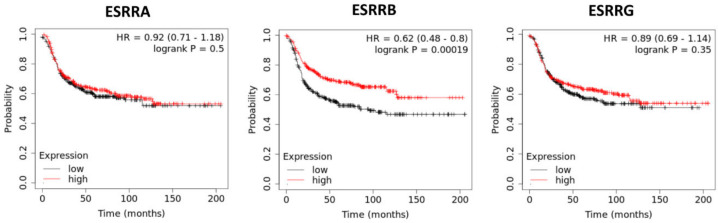
Kaplan–Meier analyses of the impact of ERR mRNA expression on relapse-free survival (RFS) of 879 patients with basal-like TNBC using the integrated microarray and clinical data KMplotter platform [56]. Indicated are the genes coding for ERRα, β, and γ.

When we performed a survival analysis with regard to the basal-like intrinsic subgroup [1] of TNBCs using the integrated microarray and clinical data Kaplan–Meier plotter platform (https://kmplot.com) [56] to examine the impact of ERR mRNA expression on survival of 879 patients with basal-like TNBC, we found a notable association of high ERRβ mRNA levels with prolonged RFS (upper quartile survival of the low expression cohort: 17.23 months, but 34 months in the high expression cohort), (HR = 0.62 (0.48–0.8), log rank *p* = 0.00019). ERRβ did not significantly affect OS of these patients, presumably due to the lower case numbers analyzed. Neither ERRα nor ERRγ mRNA levels were associated with RFS or OS of basal-like TNBCs (Figure 4).

**Figure 5:**
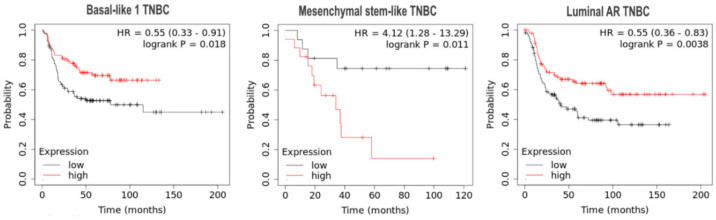
Kaplan–Meier analyses of the impact of ERRβ mRNA expression on relapse-free survival (RFS) of patients with the indicated TNBC subtypes identified by Lehmann et al. [8] using the integrated microarray and clinical data KMplotter platform [56]. The subtypes not indicated here did not show any association between ERRβ expression and survival.

Performing a survival analysis with regard to the 6 TNBC subgroups identified by Lehmann et al. [8], we observed a significant association of high ERRβ mRNA levels with longer RFS in the basal-like 1 TNBC subgroup (*n* = 239) (upper quartile survival of the low expression cohort: 16 months, survival of the high expression cohort: 39 months, HR = 0.55, log rank *p* = 0.018) (Figure 5). Additionally, ERRβ mRNA levels were associated with longer RFS in the luminal AR (LAR) TNBC subgroup (*n* = 276) (HR = 0.55, log rank *p* = 0.0038), but associated with short RFS in the mesenchymal stem-like (MSL) TNBC subgroup (*n* = 115) (HR = 4.12, log rank *p* = 0.011). Neither ERRα nor ERRγ expression was associated with survival in one of the 6 TNBC Lehmann/Pietenpol subtypes [8].

## 4. Endocrine Therapy of TNBC Targeting Estrogen Signaling

Today, chemotherapy is the state-of-the-art approach for treatment of TNBC. However, as mentioned above, there is some evidence that therapy strategies targeting ERα-independent estrogen signaling might be efficient in TNBC. The idea to activate ERβ as an endocrine strategy to treat TNBC primarily emerged from various studies showing that activation of this receptor by E2 or specific agonists such as ERB-041, WAY200070, or FERb 033 was able to notably reduce TNBC cell growth, arrest cell cycle at the G1 phase, block cell colony formation, inhibit TNBC cell invasiveness, and reduce tumor size in mice xenografts [41,43,44,84], as well as from other studies demonstrating ERβ to exert antitumoral actions.

Consequently, in 2019, the use of E2 in treating ERβ-positive TNBC patients was approved for clinical trials, and the phase II trial “Therapeutic Targeting of ER Beta in Triple Negative Breast Cancer” has been launched (ClinicalTrials.gov Identifier: NCT03941730). However, it remains to be examined whether the type of endocrine intervention in TNBC should be based on E2 treatment to activate the tumor-suppressing functions of ERβ, or on the use of antiestrogens or aromatase inhibitors, since single studies reported faslodex to have potent growth-inhibitory effects on ERβ-positive TNBC and tamoxifen to exert beneficial actions in ERβ-positive TNBC with mutant TP53 [39,45]. Aromatase inhibitor letrozole has been reported to decrease the frequency of brain metastases in TNBC [37].

Estrogen-related receptors (ERRs) and GPER-1 are receptors known to activate or modulate estrogen signaling in TNBC and thus are additional potential targets for therapy of this cancer entity. With regard to ERRs, ERRα in particular has been considered to be a promising target for treatment of TNBC. A recent study reported pharmacological inhibition by inverse ERRα agonist XCT-790 to suppress the growth of TNBC cells through ROS generation and induction of mitochondrial-related apoptosis in vitro and in vivo [85]. Another new synthetic inverse agonist of ERRα, LingH2-10, was reported to inhibit migration of TNBC cells and to notably suppress growth of TNBC MDA-MB-231 cell xenografts [86]. A synthetic ERRα ligand named compound 11 potently inhibited ERRα’s transcriptional activity and inhibited the migration of TNBC breast cancer cells. In vivo, compound 11 demonstrated a strong inhibitory effect on the growth of TNBC xenografts (MDA-MB-231), reducing the tumor growth by 40.9% [87]. Although the exact role of GPER-1 in TNBC is still controversial, activation of this receptor by its specific agonist G-1 was reported to exert antitumoral effects on TNBC cells. In a recent study, G-1, which triggered activation of GPER-1, was shown to suppress migration and invasion of TNBC cells by inhibition of epithelial mesenchymal transition (EMT) via NF-κB signals; these results could be confirmed using MDA-MB-231 tumor xenografts in nude mice [70]. In another study, GPER-1 ligand G-1 was able to suppress migration and angiogenesis of TNBC cells via inhibition of NF-κB/IL-6 signals in vitro and in vivo [71]. 

In conclusion, the evidence demonstrating that the activation of ERβ (e.g., by E2) and the pharmacological targeting of ERRs (particularly ERRα) and GPER-1 is able to exert antitumoral actions on TNBC cells strongly suggests that endocrine therapy options directed against these targets should be considered for treatment of patients with TNBC. In addition to the mentioned ongoing phase 2 study examining the effect of E2-triggered ERβ activation in TNBC, further in vivo studies and clinical trials are necessary to elucidate the eligibility of ERRs or GPER-1 as targets for endocrine treatment of this cancer entity.
